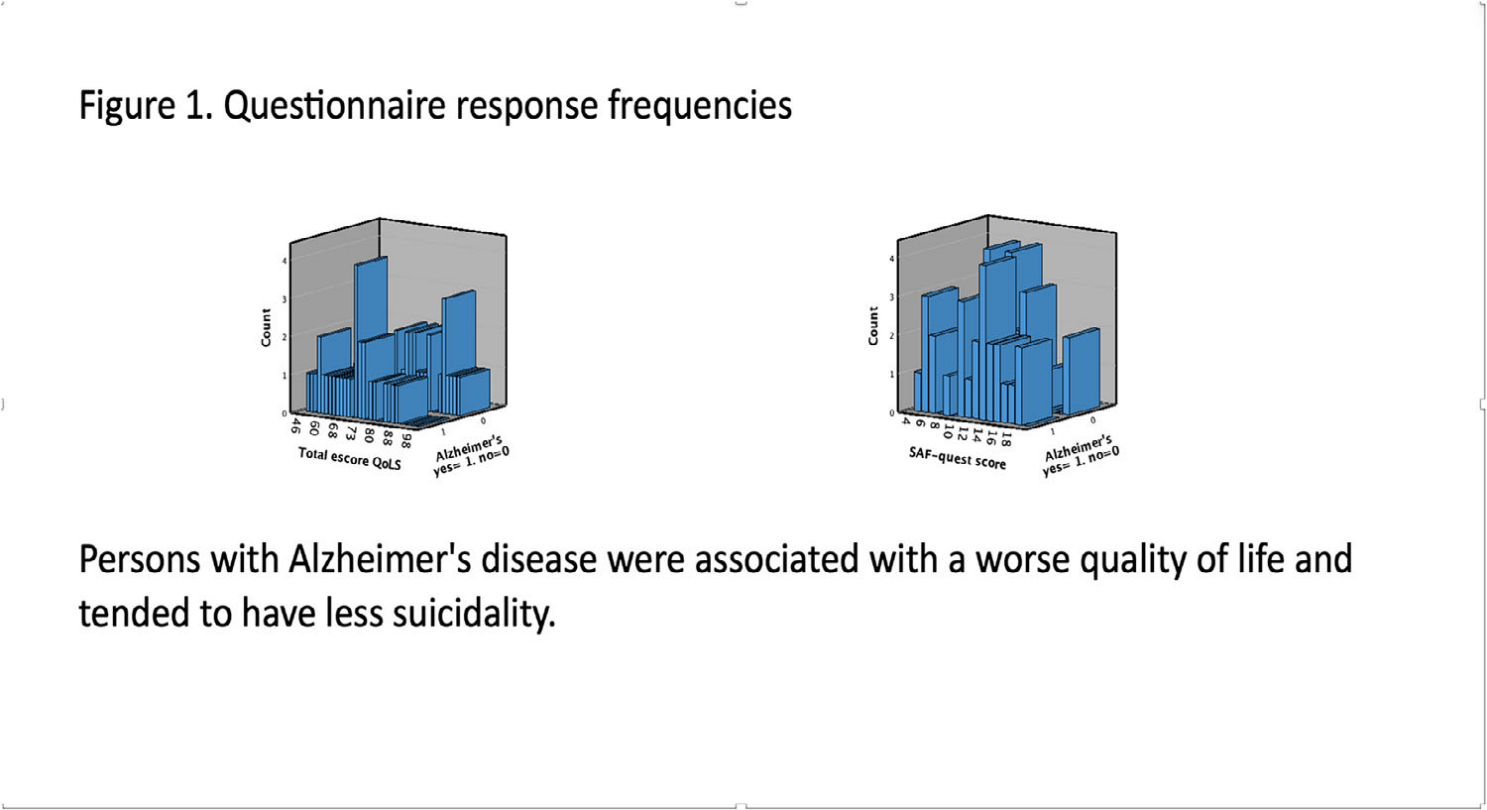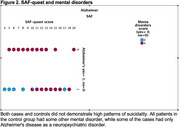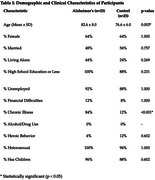# Suicidal Behavior in Alzheimer's Disease: Preliminary Study

**DOI:** 10.1002/alz70857_097455

**Published:** 2025-12-24

**Authors:** Juliano Flavio Rubatino Rodrigues

**Affiliations:** ^1^ Consultório Médico, MarIlia, São Paulo, Brazil; Faculdade de Medicina de São José do Rio Preto, São José do Rio Preto, São Paulo, Brazil

## Abstract

**Background:**

Suicidal behavior presents a significant dilemma in the context of Alzheimer's disease. Numerous ethical discussions have emerged regarding euthanasia for patients suffering from neurodegenerative conditions, and research indicates an elevated incidence of suicide in the early stages of dementia. However, there remains a gap in knowledge concerning the historical prevalence of suicidal ideations or attempts among individuals diagnosed with Alzheimer's disease. This study aims to investigate the historical patterns of suicidal behavior and the associated factors across the lifespan in patients with Alzheimer's disease.

**Methods:**

This study is an excerpt from case‐control research, where the sample was calculated at 150 participants, 75 for the case group and 75 for the control group. Here, the descriptive statistics of the first third of the sample, 50 participants, were made.

**Results:**

24% of control group participants had suicidal ideation throughout life and 12% of cases (OR for suicidal ideation = 0.432 [CI 95%: 0.095‐1.966]). 8% of control group participants attempted suicide throughout life and 4% of cases (OR for suicide attempts = 0.479 [CI 95%: 0.41‐5.652]). Persons with Alzheimer's disease were associated with a worse quality of life and tended to have less suicidality. (Figure 1 and 2, Table 1)

**Conclusions:**

It appears that suicidal behavior is inversely related to the risk of developing suicide. The odds ratio data demonstrate the need for a larger sample size to determine whether there is a difference in the history of suicide throughout the lives of people with Alzheimer's disease and the general population.